# Costs and health burden of surgical site infections and anastomotic leakage in major abdominal surgery

**DOI:** 10.1093/bjsopen/zrag111

**Published:** 2026-09-29

**Authors:** Nathan Bontekoning, Hannah Groenen, Stijn W de Jonge, Markus W Hollmann, Marja A Boermeester, Marcel G W Dijkgraaf, Niels Wolfhagen, the EPO2CH Study Group, Q J J Boldingh, W J Bom, J C G Scheijmans, L R C W van Lonkhuijzen, A Demirkiran, O E van Geffen, E B Kluyver, E R Hendriks, J-P Hering, D J A Sonneveld, J A B van der Hoeven, B M F van der Leeuw, L M Posthuma

**Affiliations:** Department of Surgery, Amsterdam UMC, University of Amsterdam, Amsterdam, the Netherlands; Amsterdam Gastroenterology Endocrinology & Metabolism, Amsterdam, the Netherlands; Department of Surgery, Amsterdam UMC, University of Amsterdam, Amsterdam, the Netherlands; Amsterdam Gastroenterology Endocrinology & Metabolism, Amsterdam, the Netherlands; Department of Surgery, Amsterdam UMC, University of Amsterdam, Amsterdam, the Netherlands; Amsterdam Gastroenterology Endocrinology & Metabolism, Amsterdam, the Netherlands; Department of Anaesthesiology, Amsterdam UMC, University of Amsterdam, Amsterdam, the Netherlands; Department of Surgery, Amsterdam UMC, University of Amsterdam, Amsterdam, the Netherlands; Amsterdam Gastroenterology Endocrinology & Metabolism, Amsterdam, the Netherlands; Department of Surgery, Amsterdam UMC, University of Amsterdam, Amsterdam, the Netherlands; Epidemiology and Data Science, Amsterdam UMC, University of Amsterdam, Amsterdam, the Netherlands; Methodology, Amsterdam Public Health, Amsterdam, the Netherlands; Department of Surgery, Amsterdam UMC, University of Amsterdam, Amsterdam, the Netherlands; Amsterdam Gastroenterology Endocrinology & Metabolism, Amsterdam, the Netherlands

## Abstract

**Background:**

Surgical site infection (SSI) is a common postoperative complication. SSI, including anastomotic leakage (AL), is a burden for patients and leads to increased costs, although insights as to the true costs from prospective data are limited. This study aimed to provide an overview of the impact of SSI and AL specifically on total societal costs and the quality of life (QoL) of patients.

**Method:**

This is a secondary analysis from the EPO_2_CH trial conducted between 2016 and 2020, which randomized patients scheduled for elective abdominal surgery with incisions > 5 cm to either standard care or standard care plus a bundle aiming to reduce SSI. Prospectively collected clinical data from the EPO_2_CH trial were used to ascertain the occurrence of SSI (superficial/deep/organ space and AL). The costs and QoL of patients with *versus* without SSI within 90 days after surgery were evaluated. The primary outcomes were costs (€) and (loss of) health utility, measured as quality-adjusted life-years (QALYs). QoL data were collected using patient questionnaires (EQ-5D™-3L). Unit costs were based on national guidelines for health economic evaluations and locally available unit costs of care.

**Results:**

Overall, 1119 of 1777 patients were analysed. Costs in the SSI and non-SSI groups were €53 562 (bias-corrected and accelerated (BCa) 95% confidence interval (c.i.) 45 356 to 68 600]) and €23 969 (BCa 95% c.i. 22 961 to 25 075), respectively, with a mean difference of €29 592 (BCa 95% c.i. 21 206 to 45 674). The greatest mean difference was observed between patients with AL and patients without SSI (€56 401; BCa 95% c.i. 39 487 to 90 892). The maximum attainable mean QALY score over 3 months was 0.25. The mean(standard deviation) QALY score in SSI and non-SSI patients was 0.171(0.050) and 0.205(0.037), respectively, with a mean difference of −0.035 (BCa 95% c.i. −0.042 to −0.029). This corresponds to a loss of 12.78 days in perfect health over the 3-month period.

**Conclusion:**

Patients with SSI, regardless of its type, had substantially higher healthcare costs and lower QoL after major abdominal surgery than patients without SSI.

## Teaser

This study quantifies the true societal costs and health-related quality-of-life impact of surgical site infections and anastomotic leakage following major abdominal surgery using prospectively collected data from a multicentre randomized clinical trial. Surgical site infections were associated with substantially higher healthcare and societal costs and a significant loss in quality-adjusted life years, with the greatest burden observed in patients with anastomotic leakage. These findings demonstrate that the economic and health impacts of these complications are considerably greater and more persistent than previously estimated.

## Introduction

Surgical site infection (SSI) is the most common postoperative complication, resulting in excess morbidity and mortality and increased costs^1–3^. Leakage of the surgical anastomosis is a severe form of SSI^4^. SSIs are deemed multifactorial, with various interventions shown to be are effective in preventing them^5–7^, whereas many others are still under investigation. New preventive measures may increase healthcare costs. To determine clinical benefit for patients and evaluate the cost-effectiveness of new interventions, reliable information on the relationships between SSIs and cost and patients’ quality of life (QoL) is vital. High-quality data are lacking because current estimations on the cost of SSIs are not based on prospectively collected detailed data and do not include total societal costs^8–10^. As a result, estimations of the implications of SSIs vary widely and uncertainty remains^9,10^.

The EPO_2_CH trial studied a set of interventions, collectively forming the EPO_2_CH bundle for the prevention of SSI in major abdominal surgery^11,12^. The bundle did not show any significant effects in reducing SSI^13^, but the trial did collect comprehensive cost and QoL data.

Using EPO_2_CH trial data, the aim of this study was to evaluate the economic and QoL burden of SSIs (including anastomotic leakage) following major abdominal surgery in a high-income setting.

## Methods

This study was an analysis of prospectively gathered data from the EPO_2_CH trial to assess the impact of SSI on costs and QoL. The study was performed in accordance with the Dutch guideline for health economic evaluations and is reported according to the CHEERS guideline^14,15^.

The EPO_2_CH trial was approved by the Amsterdam UMC Medical Ethics Committee (Reference 2015_121, approval date 22 July 2015), as well as the institutional review boards of all seven participating centres. All patients provided informed consent.

### EPO_2_CH trial

The design, statistical analysis plan, and results of the EPO_2_CH trial have been published elsewhere^11–13^. The trial was an open-label, pragmatic, randomized, controlled, parallel-group multicentre trial conducted between 2016 and 2020. Patients scheduled for elective abdominal surgery with abdominal incisions larger than > 5 cm were included. Patients were randomized to standard care with or without the EPO_2_CH bundle added. Pragmatic standard care was defined as that specified by the treating physician. The EPO_2_CH bundle consists of intraoperative high fractional inspired oxygen; intraoperative goal-directed fluid therapy; active perioperative warming; strict glucose control and treatment of hyperglycaemia (> 10 mmol/l) in diabetic and non-diabetic patients; and wound irrigation using an aqueous antiseptic before closure.

The primary outcome of the EPO_2_CH trial was the proportion of patients with SSI within 30 days after surgery, as defined by the Centers for Disease Control and Prevention^16^. Secondary outcomes included, among others, SSI within 90 days after surgery, type of SSI (that is, superficial, deep, organ space or anastomotic leakage), health utility scores, and costs (healthcare and productivity loss costs)^17^. Outcome data were prospectively gathered by treating physicians during routine postoperative care and recorded in either the complication register or patients’ medical records.

No significant effect of the bundle on SSI incidence was found; hence, no formal cost-effectiveness analysis was performed.

### Study population

Patients of the original trial were included if complete costs from the electronic health record, data on out-of-hospital costs, and any data regarding health utility were available. Patients who did not return any health utility questionnaire were excluded.

### Outcomes of interest

The primary outcomes of the present study were the cost, measured in €, and quality-adjusted life-years (QALYs) of patients with and without SSI within 90 days after surgery. Secondary outcomes were utility scores at specific follow-up time points. Costs include healthcare costs and productivity loss costs, and were estimated using a mix of bottom-up and top-down microcosting methodology^18,19^. The time horizon was 90 days after surgery.

SSI were categorized as superficial SSI, deep SSI, organ space SSI without anastomotic leak and anastomotic leak.

### Cost components

All costs were considered from a societal perspective. In-hospital costs cover the cost of the surgery, inpatient stay on the intensive care unit (ICU) and/or wards, diagnostic and therapeutic procedures, emergency care, readmissions, outpatient consultations with surgeons and/or other specialties, and consultations with physiotherapist or other healthcare professionals.

To analyse these costs comprehensively, volume data on in-hospital resources were extracted from the electronic health record for each patient. These data were then grouped according to the respective care category.

Data on out-of-hospital resource use were gathered using an adjusted version of the institute for Medical Technology Assessment (iMTA) Medical Consumption Questionnaire (iMCQ)^20^ at 90 days after surgery. The employer costs reflected losses of productivity resulting from both absenteeism and presenteeism (reduced productivity due to illness despite being present at work). These costs were estimated using the iMTA Productivity Cost Questionnaire (iPCQ)^21^ at 90 days. Productivity losses were based on the friction cost method, with the study observation period serving as the actual friction period^22^.

No data on patients’ healthcare-related out-of-pocket expenses (including informal care, over-the-counter medications, and healthcare-related travel costs) were calculated or gathered.

### Unit costing and costs

The unit costing of healthcare resources was performed according to the most recent guidelines for national healthcare costing for economic evaluations in the Netherlands^15^. That guideline provides reference unit costs for common resources like a visit to the outpatient clinic or an overnight stay in the ward of a general hospital. If reference unit costs were unavailable or deemed inappropriate, unit costs on locally available unit costs of care or on passer-by tariffs were used. All costs are expressed in euros (€) for the base year of 2022. If required, price indexing was applied if the unit costs stemmed from other base years, using yearly consumer price indices for the Netherlands^23^. Mean unit costs per category are presented in *Table S1*.

### Health outcomes

Health utility was measured using the 3-level EQ-5D™ (EQ-5D-3L) questionnaire^24^ at 10, 30, 60 and 90 days after surgery. Health status profiles on the EQ-5D™-3L were converted to health utility scores based on the Dutch general population-based tariffs, theoretically ranging from a score of −0.33 to 1 for perfect health^25^. In the absence of baseline measurements, the health utility score at day 10 for the preceding 9 days was used. For the remainder of the time horizon, linear interpolation between successive health utility assessments was applied. QALYs were calculated for each participant from the area under the curve of these measurements. The maximum QALY score is 0.25, given the 3-month time horizon.

### Statistical analysis

Costs were calculated for individual patients as the sum of the products of the amount of healthcare resources used and their respective unit costs. Mean units per cost category are presented where possible. Costs and differences between SSI and non-SSI patients are shown as mean values with their bias-corrected and accelerated (BCa) 95% confidence interval (c.i.).

Missing health utility scores from patients who filled out at least one EQ-5D™-3L, were imputed if missing. Missing data were assessed for missingness (completely) at random and, if so, were handled with multivariate imputation by chained equations with predictive mean matching. The predictors used were all preoperative baseline characteristics, procedure characteristics (including surgery indication, surgery type, hospital type, SSI occurrence, length of hospital stay) and the non-missing questionnaires; these were used to estimate the missing data. Fifty rounds of imputation were performed. The results of individual missing questions were estimated. Health utility and QALY scores were calculated from these questions. Data from imputed data sets were pooled. QALY and utility scores are shown as the mean with standard deviation (s.d.), with the mean difference (95% c.i.) when available.

Planned subgroup analyses were performed to assess the impact of SSI severity. Costs and the QALY of patients with superficial, deep, organ space, and anastomotic leakage SSI were compared to those of non-SSI patients. Other subgroup analyses for QALY were stratified for surgery for either a malignant or benign disease, and cost analysis was stratified by hospital type, distinguishing between academic and non-academic institutions. In addition, an unplanned exploratory analysis was performed to assess differences in specific cost categories between academic and non-academic hospitals, exploring potential variation in resource use and associated costs between hospital types.

The inverse probability of treatment weighting (IPTW) was applied for sensitivity analyses to adjust for potential confounding, to check whether the total incremental costs or decrease in QALY (for each SSI severity) could be attributed solely to the occurrence of an SSI. By using weighting based on the inverse of the propensity score, the imbalance in covariates was reduced. A standardized difference of < 10% was deemed to indicate appropriate balance. Propensity scores were estimated using the following baseline variables selected *a priori* based on their potential influence on both the occurrence of SSI and the outcomes, informed by clinical reasoning and existing literature: age, sex, body mass index, American Society of Anesthesiologists grade, smoking status, diabetes, chronic obstructive pulmonary disease, cardiovascular disease other than hypertension, a history of abdominal surgery, type of hospital, the indication for surgery, the type of surgery, wound classification, and operation time. Covariate balance for the different weighting analyses are presented using Love plots. E values were calculated to assess possible unmeasured confounding.

Two-tailed *P <* 0.05 was considered statistically significant. All analyses were conducted using R version 4.3.2 (R Foundation for Statistical Computing, Vienna, Austria).

## Results

### Participant characteristics

In the EPO_2_CH trial, 1777 participants were analysed. Data from the electronic health records regarding care provided was available for 1728 participants, with 1351 participants completing the out-of-hospital cost questionnaire (iMCQ) and 1269 completing the productivity questionnaire (iPCQ). Data regarding health utility were available for 1605 participants.

Combining these data sets resulted in a data set with complete data for 1119 patients (*Fig. S1*). Of these patients, 210 were diagnosed with an SSI within the 90-day follow-up period. Of the 61 patients who experienced an anastomotic leak, 12 underwent hepatopancreatobiliary procedures. Baseline characteristics of the study population are presented in *Table 1*. Baseline characteristics of included and excluded patients are presented in *Table S2*. Several significant differences were observed between groups.

**Table 1:** Patient and procedure characteristics

	SSI (*n* = 210)	Non-SSI (*n* = 909)	P*
**Preoperative baseline characteristics**			
Age (years), mean(s.d.)	62.8 (12.0)	63.5 (12.4)	0.462
Sex			0.162
Female	106 (50.5%)	510 (56.1%)	
Male	104 (49.5%)	399 (43.9%)	
BMI (kg/m^2^), mean(s.d.)	27.5 (5.5)	26.1 (4.8)	< 0.001
ASA grade			0.104
I	17 (8.1%)	125 (13.7%)	
II	142 (67.6%)	600 (66%)	
III	49 (23.3%)	178 (19.6%)	
IV	1 (0.5%)	5 (0.6%)	
Unknown	1	1	
Current smoker	29 (13.8%)	124 (13.6%)	0.958
Diabetes	34 (16.2%)	109 (12%)	0.127
COPD	16 (7.6%)	57 (6.3%)	0.577
CVD other than hypertension	33 (15.7%)	114 (12.5%)	0.266
History of abdominal surgery	121 (57.6%)	397 (43.7%)	< 0.001
**Procedural characteristics**			
Type of hospital			0.056
Academic	105 (50%)	372 (40.9%)	
Non-academic	105 (50%)	537 (59.1%)	
Indication for surgery			< 0.001
Benign	84 (40%)	203 (22.3%)	
Malignancy	126 (60%)	705 (77.6%)	
Surgery type			< 0.001
Colorectal	116 (55.2%)	561 (61.7%)	
General	36 (17.1%)	94 (10.3%)	
Gynaecological	9 (4.3%)	132 (14.5%)	
Hepatopancreatobiliary	40 (19%)	96 (10.6%)	
Upper gastrointestinal	9 (4.3%)	26 (2.9%)	
Wound classification			0.002
Clean	14 (6.7%)	48 (5.3%)	
Clean/contaminated	178 (84.8%)	826 (90.9%)	
Contaminated	10 (4.8%)	30 (3.3%)	
Dirty	8 (3.8%)	5 (0.6%)	
Operation time (h), mean(s.d.)	3.8 (1.9)	3.0 (1.4)	< 0.001

Values are *n* (%) unless otherwise stated. SSI, surgical site infection; s.d., standard deviation; BMI, body mass index; ASA, American Society of Anesthesiologists; COPD, chronic obstructive pulmonary disease; CVD, cardiovascular disease; h, hours. *P values were calculated using Student’s *t* test for continuous variables and the χ^2^ test for categorical variables.

For the health utility scores, missing questionnaire data were imputed. Overall, 84.4% (944 of 1119) of included patients provided complete health utility observations. The magnitude of imputed data per questionnaire is provided in *Table S3*. There was no missingness in the cost data.

### IPTW analyses

*Figure S2* summarizes the balance for the covariates before and after IPTW for the entire data set (*Figure S2a*), and the balance for the respective data sets used in subgroup analyses with only superficial (*Figure S2b*), anastomotic leakage (*Figure S2c*) and organ space (*Figure S2d*) SSI patients, respectively. No IPTW analysis was performed on deep SSI due to limited data. E values indicating the level of possible unmeasured confounding for the IPTW analyses are presented in *Table S4*.

### In-hospital costs

In-hospital costs were €24 714 (BCa 95% c.i. 17 497 to 40 561) higher for patients with an SSI (*Table S5*). This cost increase occurred over all domains, but was dominated by the increased cost of general ward and ICU stays. Only the cost of pathology examinations did not differ between the SSI and non-SSI groups.

### Out-of-hospital costs

The increase in total out-of-hospital costs was €2214 (BCa 95% c.i. 1232 to 3988) for SSI patients compared with non-SSI patients. The largest portion of this difference was due to home nursing costs, with a mean difference of €1413 (BCa 95% c.i. 739 to 2463) between the SSI and non-SSI groups (*Table S6*). No significant differences were found in costs related to social work, homeopathy, company physician services, or housekeeping. All other out-of-hospital cost categories showed significantly higher mean costs for SSI patients. A detailed breakdown, including mean units or hours of care used per patient, is provided in *Table S6*.

### Productivity losses

A mean difference of €2664 (BCa 95% c.i. 457 to 10 317) was found in productivity losses between the SSI and non-SSI groups among all patients and €3887 (BCa 95% c.i. 250 to 16 262) among working individuals. Productivity loss details are outlined in *Tables S7* and *S8*.

### Total costs

The mean total cost for SSI patients was €53 562 (BCa 95% c.i. 45 356 to 68 600), compared with €23 969 (BCa 95% c.i. 22 961 to 25 075) for non-SSI patients, a cost difference of €29 592 (BCa 95% c.i. 21 206 to 45 674; *Table 2*). Adjustment for differences in patient and procedure characteristics through IPTW showed comparable cost differences between the two groups.

**Table 2:** Total costs per category

	Mean costs (€)			
	SSI	Non-SSI	Difference	Weighted difference
In-hospital*	43 186 (35 731, 58 532)	18 472 (17 835, 19 194)	24 714 (17 497, 40 561)	23 587 (14 938, 58 604)
Out-of-hospital†	3250 (2263, 5013)	1036 (827, 1333)	2214 (1232, 3988)	1845 (890, 5645)
Productivity	7126 (4985, 14 012)	4462 (3859, 5220)	2664 (457, 10 317)	2181 (447, 32 965)
Overall costs	53 562 (45 356, 68 600)	23 969 (22 961, 25 075)	29 592 (21 206, 45 674)	27 613 (18 671, 67 848)

Values in parentheses are bias-corrected and accelerated 95% confidence intervals. *Detailed non-weighted in-hospital costs by category are provided in *Table S5*. †Detailed non-weighted out-of-hospital costs by category are provided in *Table S6*. SSI, surgical site infection .

### Cost subgroup analyses

Stratified analysis on SSI severity showed that associated costs are higher for every type of SSI compared with patients without an SSI. Cost increased with the severity of infection. The greatest mean difference was seen between a patient experiencing anastomotic leakage and a non-SSI patient: €56 401 (BCa 95% c.i. 39 487 to 90 892). Adjustment for differences in patient and procedure characteristics through IPTW resulted in comparable cost differences between the two groups. *Table 3* shows the full subgroup analysis.

**Table 3:** Costs by type of SSI

Type of SSI*	No. of patients	Mean costs (€)			
		SSI	Non-SSI (*n* = 909)	Difference	Weighted difference
All SSI	210	53 562 (45 356, 68 600)	23 969 (22 961, 25 075)	29 592 (21 206, 45 674)	27 613 (18 671, 67 848)
Superficial SSI	81	33 197 (27 499, 50 043)	23 969 (22 961, 25 075)	9228 (3487, 27 756)	8417 (3797, 70 212)
Deep SSI	16	39 553 (34 198, 46 078)	23 969 (22 961, 25 075)	15 584 (9797, 22 122)	NE
Organ space SSI (no AL)	50	59 528 (40 742, 125 590)	23 969 (22 961, 25 075)	35 559 (16 794, 101 960)	32 863 (11 799, 312 900)
Anastomotic leakage	61	80 371 (63 661, 115 876)	23 969 (22 961, 25 075)	56 401 (39 487, 90 892)	53 930 (31 577, 225 350)

Values in parentheses are bias-corrected and accelerated 95% confidence intervals. *For two patients, no SSI severity was reported. SSI, surgical site infection; AL, anastomotic leakage; NE, not estimable (due to small subgroup and extreme weights).

The associated mean difference was greater in non-academic hospitals (€41 819) than in academic hospitals (€16 499). In the explorative analyses, a large difference in ICU stay costs was found in non-academic hospitals (€16 629; BCa 95% c.i. 6248 to 41 508). Detailed costs per hospital type are presented in *Tables S9–S11*.

### QoL outcomes

Patients with an SSI reported lower QoL in the first 3 months after surgery. Of the maximum attainable score of 0.25 in the 3-month period, a mean(s.d.) QALY of 0.169(0.052) was calculated for patients with an SSI, compared with 0.204(0.038) for non-SSI patients, with a mean difference of −0.035 (BCa 95% c.i. −0.042 to −0.029). This corresponds to a mean loss of 12.78 days in perfect health over the 3-month period. The mean health utility scores on days 10, 30, 60 and 90, together with the maximum mean QALY score over 3 months, are shown in *Fig. 1*. Details on QALY are presented in *Table 4*, and mean health utility scores are reported in *Table S12*.

**Figure 1:**
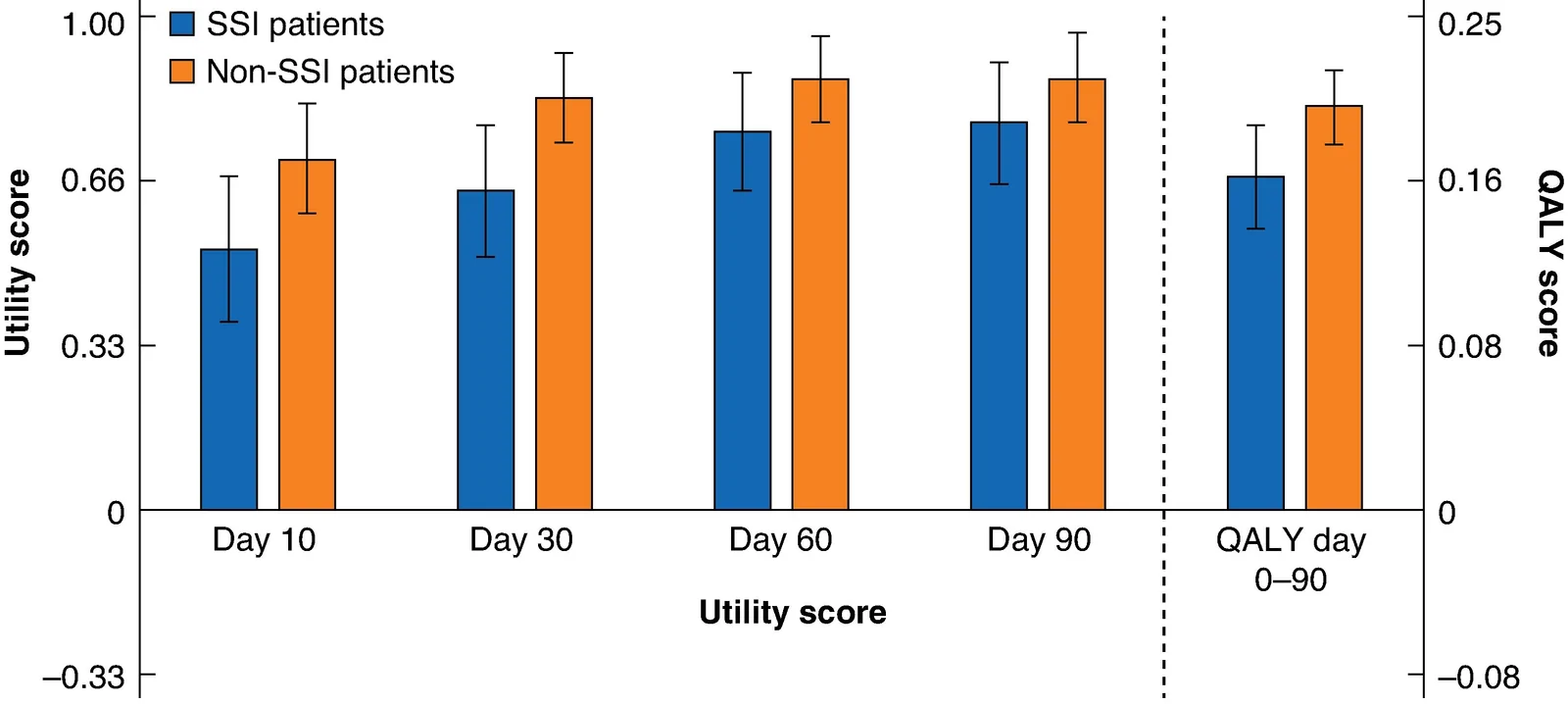
Mean utility and QALY scores for SSI and non-SSI patients over a 90-day period Error bars indicate the standard deviation. Utility scores range from −0.33 to 1 (perfect health); QALY scores range from −0.08 to 0.25 (perfect health). Axis increments have been adjusted for readability, and tick marks are not exact multiples. QALY, quality-adjusted life years; SSI, surgical site infection.

**Table 4:** Health utility over 90 days (QALY) by type of SSI

Type of SSI*	SSI		Non-SSI mean(s.d.) QALY (*n* = 909)	Mean difference	Weighted mean difference
	No. of patients	Mean(s.d.) QALY			
All SSI	210	0.169(0.052)	0.204(0.038)	−0.035 (−0.042, −0.029)	−0.031 (−0.038, −0.023)
Superficial SSI	81	0.183(0.044)	0.204(0.038)	−0.021 (−0.030, −0.012)	−0.018 (−0.027, −0.008)
Deep SSI	16	0.170(0.043)	0.204(0.038)	−0.035 (−0.054, −0.017)	NE
Organ space SSI (no anastomotic leakage)	50	0.161(0.061)	0.204(0.038)	−0.042 (−0.053, −0.031)	−0.033 (−0.050, −0.016)
Anastomotic leakage	61	0.155(0.053)	0.204(0.038)	−0.049 (−0.059, −0.039)	−0.048 (−0.063, −0.034)

Values in parentheses are 95% confidence intervals unless otherwise stated. The maximum achievable QALY score over the 3-month time period was 0.25. *For two patients, no SSI severity was reported. QALY, quality-adjusted life years; SSI, surgical site infection; s.d., standard deviation; NE, not estimable (due to small subgroup and extreme weights).

### QALY subgroup analyses

Significant differences in QALY between SSI and non-SSI patients were found for both benign and malignancy surgical indications, as well as for all different types of SSI. Full subgroup analyses outcomes are presented in *Table S13*.

## Discussion

This study evaluated the cost and health impacts of SSI and anastomotic leakage after major abdominal surgery, using data from the EPO_2_CH trial. SSI led to higher costs with a mean difference in total costs of €29 592 (BCa 95% c.i. 21 206 to 45 674), with the lowest cost difference for superficial SSI (€9228; BCa 95% c.i. 3487 to 27 756) and the highest difference for anastomotic leakage (€56 401; BCa 95% c.i. 39 487 to 90 892). Increased costs were accompanied by a loss in QALY from the maximum achievable QALY score over the 90-day follow-up period, with the highest loss in QALY seen for anastomotic leakage. Adjustment for patient and procedural characteristics yielded similar results.

Estimates of previous studies on cost attributable to SSI and anastomotic leakage vary widely^8,10,26–28^. Most of these studies rely on retrospective analysis of registry data and are prone to under-reporting, with limited data on out-of-hospital costs and productivity losses and no differentiation based on SSI severity^10,28,29^. For example, a previous study^10^ estimated SSI cost across a wide range of surgical procedures based on retrospective reimbursement data from German hospitals and found that, on average, SSIs cost €9968. A recent post hoc analysis^8^ of data collected prospectively during the RECIPE trial found that SSI after major surgery led to €5540 in additional costs. That study was a single-centre academic study, anastomotic leakage was not included in the analysis, and follow-up was limited to 30 days^8^. Neither study^8,10^ included out-of-hospital costs and productivity losses. A systematic review^26^ of the cost of anastomotic leakage concluded that data from a societal perspective on the cost of anastomotic leakage is scarce and that societal burden is under-reported. Reported costs ranged from €2250 up to €83 633, with only retrospective cohort studies included in that review^26^.

A review evaluating loss of QoL due to anastomotic leakage found that existing studies are heterogeneous regarding their methodology, follow-up, and results, but indicate considerable loss of QoL up to 1 year after surgery^30^. Two studies reported QALY data after anastomotic leakage at 3 months with conflicting results^31,32^. In contrast with previous studies, the present study provides comprehensive prospective data from a multicentre randomized clinical trial including in-hospital costs, out-of-hospital costs, productivity losses and QALY data, with the latter being the first such data on SSI to be published. The findings of this study indicate that costs attributable to SSI are higher than previously estimated and that the effects of SSI on QoL persist long after treatment. Although more severe infections led to higher costs and lower QALY scores, importantly even superficial infections led to considerable costs and loss of QoL. In-hospital costs were the largest contributor to cost, and resulted predominantly from longer general ward and ICU stays, as well as additional operative procedures. This is contrasted by modest additional costs from home nursing care after discharge. The costs of SSI were higher in non-academic hospitals, largely driven by more and longer ICU stays compared with patients with an SSI in academic hospitals. Potentially this could be explained by the fact that in the academic centres patients stayed overnight in a postoperative anaesthesia care unit, whereas general hospitals do not have these facilities.

Together, the findings demonstrate the true economic and health impacts of SSI, providing evidence that is directly relevant for clinicians, hospital administrators, and policymakers. Quantifying both incremental cost and decrease in QALY allows a more informed evaluation of investments in preventive measures. Although the implementation of preventive strategies may incur upfront costs, the magnitude of potential savings in healthcare resources and improvement in patient QoL suggest substantial return on investment.

Several study limitations should be acknowledged. One limitation is the extent of missing data, particularly for the out-of-hospital cost (iMCQ) and productivity (iPCQ) questionnaires, which resulted in data available for only 63% of participants in the EPO_2_CH trial. Comparison of included and excluded patients revealed differences in several baseline characteristics, suggesting that missing data may not be fully random and that some degree of selection bias cannot be excluded. Furthermore, the presented data was collected prospectively in the context of a randomized clinical trial, and the cohort groups are based on the primary outcome of that trial. As a result, the cost of SSI may be confounded by patient characteristics associated with the risk of SSI and cost, such as co-morbidity or complex high-risk procedures. To account for these biases, IPTW estimates that adjusted for these potential imbalances were calculated, with comparable results found. This further strengthens the confidence in the reliability of the estimates presented in this study. Moreover, analyses were based on data from elective surgeries only, which may limit generalizability. Although outcomes and absolute costs may differ in emergency settings, the incremental costs associated with SSI treatment are expected to be broadly comparable to those observed in elective cases. Data were not collected on out-of-pocket expenses and travel costs incurred by patients, so it is possible that the true societal costs are slightly higher. Similarly, severe SSI may lead to additional costs and loss of QALY beyond the 90-day follow up. In this study, the 90-day post-surgery time horizon was chosen because SSI typically develop within 30 days of surgery^33^, with organ space SSIs, such as anastomotic leakage, often presenting within the first week and requiring immediate intervention. Although the authors acknowledge that some anastomotic leakages may even present after 90 days of follow-up in certain patient populations, it is expected that most related costs occur within this time frame and that patients would have had time to recover from most infections and any necessary interventions by the 90-day mark. Estimating health utility loss over a short period leads to deflated absolute differences compared with the actual disutility but results in a generic and comparable value for economic analysis. This study was limited to linear interpolation between measurements or the QoL analysis because no baseline measurement was taken. Although this limits comparison within groups to their respective baseline, it provides reliable estimates of differences between patients with and without SSI.

In conclusion, patients with SSI, across all severities, had substantially higher healthcare costs and lower QoL after major abdominal surgery than patients without SSI. This study presents more precise estimates of the additional costs than have been published thus far. These data are invaluable for the evaluation of resource allocation for the development and implementation of evidence-based SSI-preventive measures.

## Collaborators

Members of the EPO_2_CH Study Group are: Q. J. J. Boldingh, W. J. Bom, J. C. G. Scheijmans, L. R. C. W. van Lonkhuijzen (Amsterdam University Medical Center, Amsterdam, the Netherlands); A. Demirkiran, O. E. van Geffen, E. B. Kluyver (Rode Kruis Hospital, Beverwijk, the Netherlands); E. R. Hendriks (Tergooi Medical Center, Hilversum, the Netherlands); J.-P. Hering, D. J. A. Sonneveld (Dijklander Hospital, Hoorn, the Netherlands); J. A. B. van der Hoeven, B. M. F. van der Leeuw, (Albert Schweitzer Hospital, Dordrecht, the Netherlands); L. M. Posthuma (Amphia Hospital, Breda, the Netherlands)

## Supplementary Material

zrag111_Supplementary_Data

## Data Availability

Sharing of deidentified participant data will be considered after the publication of all secondary outcomes of this trial upon reasonable request to the corresponding author and after protocol approval and signing of a data transfer agreement. Related documents are available from the corresponding author.
