# Supplementary material for: Costs and health burden of surgical site infections and anastomotic leakage in major abdominal surgery
Source: BJS Open. 2026 Sep 29;10(5):zrag111. doi: 10.1093/bjsopen/zrag111 (PMC13621077; doi:10.1093/bjsopen/zrag111)
Supplement: Supplementary file 1 [file zrag111_supplementary_data.docx]

**True Costs and Health Burden of Surgical Site Infections and Anastomotic Leakage in Major Abdominal Surgery**

Nathan Bontekoning M.D.,1, 2 Hannah Groenen Ph.D.,1, 2 Stijn W. de Jonge Ph.D.,1, 2 Prof. Markus W. Hollmann Ph.D.,3 Prof. Marja A Boermeester Ph.D.,1, 2 Prof. Marcel G.W. Dijkgraaf Ph.D.,1,4,5* Niels Wolfhagen Ph.D.,1,2 * on behalf of the EPO2CH Study Group

**Marcel G.W. Dijkgraaf and Niels Wolfhagen contributed equally as senior author to this work.*

Members of the EPO_2_CH Study Group are co-authors of this study and are listed under the heading Collaborators.

1. Amsterdam UMC location University of Amsterdam, Department of Surgery, Meibergdreef 9, Amsterdam, The Netherlands

2. Amsterdam Gastroenterology Endocrinology & Metabolism, Amsterdam, The Netherlands

3. Department of Anaesthesiology, Amsterdam UMC, University of Amsterdam, Amsterdam, The Netherlands

4. Amsterdam UMC location University of Amsterdam, Epidemiology and Data Science, Amsterdam, The Netherlands

5. Amsterdam Public Health, Methodology, Amsterdam, The Netherlands

**Corresponding author**. Prof. M.A. Boermeester, Amsterdam UMC location University of Amsterdam, Department of Surgery, Meibergdreef 9, Amsterdam, the Netherlands. ORCID ID: 0000-0003-3890-8105

**Collaborators**

Members of the EPO_2_CH Study Group:

Q. J. J. Boldingh, W. J. Bom, J. C. G. Scheijmans, L. R. C. W. van Lonkhuijzen (Amsterdam University Medical Center, Amsterdam, The Netherlands); A. Demirkiran, O. E. van Geffen, E. B. Kluyver (Rode Kruis Hospital, Beverwijk, The Netherlands); E. R. Hendriks (Tergooi Medical Center, Hilversum, The Netherlands); J-P. Hering, D. J. A. Sonneveld (Dijklander Hospital, Hoorn, The Netherlands); J. A. B. van der Hoeven, B. M. F. van der Leeuw, (Albert Schweitzer Hospital, Dordrecht, The Netherlands); L. M. Posthuma (Amphia Hospital, Breda, The Netherlands)

**Supplementary Materials – Index**

**Supplementary Results**

| Table S1. Mean unit costs per category. | *pag. 3* |
| --- | --- |
| Figure S1. Flowchart of available participant data. | *pag. 4* |
| Figure S2a-d. Love plots for the IPTW analyses. | *pag. 5-8* |
| Table S2. Baseline comparison of included and excluded patients. | *pag. 9* |
| Table S3. Magnitude of missingness per health utility questionnaire. | *pag. 10* |
| Table S4. E-values for IPTW analyses. | *pag. 10* |
| Table S5. In-hospital costs | *pag. 11* |
| Table S6. Out-of-hospital costs | *pag. 12* |
| Table S7. Mean productivity losses for the entire cohort (including non-workers). | *pag. 13* |
| Table S8. Mean productivity losses for working individuals only. | *pag. 13* |
| Table S9. Costs per hospital type. | *pag. 14* |
| Table S10. High academic in-hospital cost categories. | *pag. 14* |
| Table S11. High non-academic in-hospital cost categories. | *pag. 15* |
| Table S12. Mean health utility scores for different timepoints after surgery. | *pag.* 16 |
| Table S13. Reported QALY in patients treated, specified for procedure indications. | *pag.* 16 |

|  | **Unit** | **(Mean) unit cost (€ 2022)** | **Source** |
| --- | --- | --- | --- |
| **In-hospital cost categories** |  |  |  |
| General Ward Stay | Day | 909 | Local rates |
| Operative Procedures* | Procedure | 4251 | Local rates |
| Blood Products | Procedure | 260 | Local rates |
| Other Treatments | Procedure | 135 | Local rates |
| Imaging Diagnostics | Procedure | 181 | Local rates |
| Clinical Chemistry and Haematology | Procedure | 4 | Local rates |
| Microbiology and Parasitology | Procedure | 38 | Local rates |
| Pathology | Procedure | 548 | Local rates |
| Other Laboratory Testing | Procedure | 53 | Local rates |
| Other Diagnostics Procedures | Procedure | 757 | Local rates |
| Intensive Care Unit Stay | Day | 3075 | Local rates |
| Intensive Care Treatments | Procedure | 483 | Local rates |
| Outpatient Clinic & Emergency Dept. | Visit | 172 | Local rates |
| Day Treatment | Day | 381 | Local rates |
| Paramedical and supporting staff | Consultation | 36 | Local rates |
| Other In Hospital Costs | NA | 93 | Local rates |
| **Out-of-hospital consultations** |  |  |  |
| Ambulance | Trip | 528 | Guideline |
| General practicioner | Visit | 31 | Guideline |
| Physiotherapist | Visit | 39 | Guideline |
| Speech therapist | Visit | 41 | Guideline |
| Psychologist | Visit | 99 | Guideline |
| Social worker | Visit | 127 | Guideline |
| Homeopathy | Visit | 68 | Dutch association of homeopathy |
| Company physician | Visit | 120 | Guideline |
| Occupational therapist | Visit | 24 | Guideline |
| Dietician | Visit | 25 | Guideline |
| Housekeeping | Visit hourly | 33 | Guideline |
| Personal care | Visit hourly | 58 | Guideline |
| Home nursing | Visit hourly | 75 | Guideline |
| **Productivity** |  |  |  |
| Production losses paid work | hourly | 40 | Guideline |
| Production losses unpaid work | hourly | 19 | Guideline |
| *Multiple operative procedures could be registered during one operation.  NA; Not applicable. | | | |

Supplementary Results

Table S1. Mean unit costs per category.

Figure S1. Flowchart of available participant data.

iMCQ; Medical Technology Assessment (iMTA) Medical Consumption Questionnaire, iPCQ; iMTA Productivity Cost Questionnaire (iPCQ), SSI; Surgical site infection.

**Figure S2a. Love plot total SSI 90 days**

Standardised mean differences with their range before (‘Original’) and after inverse probability weighting (‘Weighted’).

*Categorised variables

ASA indicates American Society of Anaesthesiologists; BMI, body mass index

**Figure S2b. Love plot superficial SSI 90 days**

Standardised mean differences with their range before (‘Original’) and after inverse probability weighting (‘Weighted’).

*Categorised variables

ASA indicates American Society of Anaesthesiologists; BMI, body mass index

**Figure S2c. Love plot anastomotic leakage 90 days**

Standardised mean differences with their range before (‘Original’) and after inverse probability weighting (‘Weighted’).

*Categorised variables

ASA indicates American Society of Anaesthesiologists; BMI, body mass index

**Figure S2d. Love plot organ space SSI 90 days**

Standardised mean differences with their range before (‘Original’) and after inverse probability weighting (‘Weighted’).

*Categorised variables

ASA indicates American Society of Anaesthesiologists; BMI, body mass index

**Table S2. Baseline comparison of included and excluded patients.**

|  | **Included (n = 1,119)** | **Excluded (n = 658)** | ***p*-value** |
| --- | --- | --- | --- |
| Preoperative baseline characteristics |  |  |  |
| Age - mean ± SD | 63.4 (12.3) | 63.5 (12.4) | 0.12 |
| Sex – no. (%) |  |  | 0.2 |
| Female | 616 (55%) | 510 (56%) |  |
| Male | 503 (45%) | 399 (44%) |  |
| BMI - mean ± SD | 26.4 (5.0) | 26.1 (4.8) | 0.3 |
| ASA grade – no. (%) |  |  | <0.001 |
| ASA I | 142 (13%) | 57 (8.7%) |  |
| ASA II | 742 (66%) | 405 (62%) |  |
| ASA III | 227 (20%) | 183 (28%) |  |
| ASA IV | 6 (0.5%) | 8 (1.2%) |  |
| Unknown | 2 | 5 |  |
| Current smoker – no. (%) | 153 (14%) | 106 (16%) | 0.2 |
| Diabetes mellitus – no. (%) | 143 (13%) | 124 (19%) | <0.001 |
| Chronic Obstructive Pulmonary Disease – no. (%) | 73 (6.5%) | 60 (9.1%) | 0.045 |
| Cardiovascular disease other than hypertension – no. (%) | 147 (13%) | 152 (23%) | <0.001 |
| History of abdominal surgery – no. (%) | 518 (46%) | 337 (51%) | 0.045 |
| Procedure characteristics |  |  |  |
| Type of hospital – no. (%) |  |  | <0.001 |
| Academic | 477 (43%) | 420 (64%) |  |
| Non-academic | 642 (57%) | 238 (36%) |  |
| Indication for surgery – no. (%) |  |  | 0.9 |
| Benign | 287 (26%) | 171 (26%) |  |
| Malignancy | 831 (74%) | 487 (74%) |  |
| Surgery type – no. (%) |  |  | <0.001 |
| Colorectal surgery | 677 (61%) | 305 (46%) |  |
| General Surgery | 130 (12%) | 106 (16%) |  |
| Gynaecologic surgery | 141 (13%) | 98 (15%) |  |
| Hepato-pancreato-biliary surgery | 136 (12%) | 137 (21%) |  |
| Upper gastrointestinal | 35 (3.1%) | 12 (1.8%) |  |
| Wound classification – no. (%) |  |  | 0.023 |
| Clean | 62 (5.5%) | 40 (6.1%) |  |
| Clean/contaminated | 1,004 (90%) | 578 (88%) |  |
| Contaminated | 40 (3.6%) | 19 (2.9%) |  |
| Dirty | 13 (1.2%) | 21 (3.2%) |  |
| Operation time (hours) – mean ± SD | 3.1 (1.5) | 3.4 (1.8) | 0.2 |
| SSI; Surgical Site Infection, BMI; Body Mass Index | | | |

**Table S3. Magnitude of missingness of health utility questionnaire data**

| Returned number of questionnaires at day | SSI n = 210 | No SSI n = 909 |
| --- | --- | --- |
| 10 (%) | 180 (85.7%) | 838 (92.2%) |
| 30 (%) | 201 (95.7%) | 880 (96.8%) |
| 60 (%) | 197 (93.8%) | 859 (94.5%) |
| 90 (%) | 209 (99.5%) | 908 (99.9%) |
| Complete case | 167 (79.5%) | 777 (85.5%) |

**Table S4. E-values for IPTW analyses.**

|  | **Weighted Difference** | | **E value** |
| --- | --- | --- | --- |
|  | **Mean cost** | **(BCa 95% CI)** |  |
| In-hospital costs^*^ | 23 587 | (14 938 to 58 604) | 3.101 |
| Out-of-hospital costs^**^ | 1845 | (890 to 5645) | 2.190 |
| Productivity costs | 2181 | (447 to 32 965) | 1.672 |
| Overall costs all SSI | 27 613 | (18 671 to 67 848) | 3.291 |
| Superficial SSI costs | 8417 | (3797 to 70 212) | 1.850 |
| Organ space SSI costs (no anastomotic leakage) | 32 863 | (11 799 to 312 900) | 3.718 |
| Anastomotic leakage costs | 53 930 | (31 577 to 225 350) | 6.335 |
| All SSI health utility | -0.031 | ( -0.038 to -0.023) | 3.248 |
| Superficial SSI health utility | -0.018 | ( -0.027 to -0.008) | 2.284 |
| Organ space SSI (no anastomotic leakage) health utility | -0.033 | ( -0.050 to -0.016) | 3.455 |
| Anastomotic leakage health utility | -0.048 | ( -0.063 to -0.034) | 5.021 |
| SSI; Surgical Site Infection, BCa; Bias-corrected and accelerated, CI; confidence interval | | | |
| * Detailed, non-weighted in-hospital costs by category are found in Table 2. | | | |
| **Detailed, non-weighted out-of-hospital costs by category are found in Supplementary Table 1 | | | |

## Table S5. In-hospital costs

|  | **SSI** | | | **non-SSI** | | | **Difference** | | |
| --- | --- | --- | --- | --- | --- | --- | --- | --- | --- |
|  | **Mean units** | **Mean cost** | **(BCa 95% CI)** | **Mean units** | **Mean cost** | **(BCa 95% CI)** | **Mean units** | **Mean cost** | **(BCa 95% CI)** |
| General Ward Stay | 18.1 | 16 475 | (14 732 to 18 918) | 7.68 | 6982 | (6701 to 7332) | 10.4 | 9494 | (7691 to 12 028) |
| Operative Procedures* | 2.42 | 8724 | (7894 to 9646) | 1.58 | 7079 | (6758 to 7437) | 0.84 | 1645 | (761 to 2651) |
| Blood Products | 0.81 | 212 | (142 to 325) | 0.16 | 40 | (32 to 51) | 0.65 | 172 | (104 to 290) |
| Other Treatments | 2.61 | 486 | (387 to 637) | 2.20 | 266 | (205 to 356) | 0.41 | 220 | (92 to 380) |
| Imaging Diagnostics | 4.08 | 754 | (645 to 894) | 1.18 | 209 | (185 to 239) | 2.90 | 546 | (432 to 689) |
| Clinical Chemistry and Haematology | 155.00 | 601 | (495 to 756) | 51.00 | 213 | (198 to 232) | 104.00 | 388 | (280 to 546) |
| Microbiology and Parasitology | 13.10 | 400 | (313 to 563) | 1.73 | 89 | (75 to 108) | 11.37 | 311 | (220 to 474) |
| Pathology | 1.59 | 866 | (792 to 946) | 1.64 | 897 | (861 to 937) | –0.05 | –31 | (–115 to 57) |
| Other Laboratory Testing | 1.65 | 105 | (65 to 176) | 0.44 | 20 | (13 to 32) | 1.21 | 85 | (45 to 153) |
| Other Diagnostics Procedures | 1.44 | 1039 | (760 to 1419) | 0.59 | 457 | (356 to 589) | 0.85 | 582 | (269 to 974) |
| Intensive Care Unit Stay | 3.12 | 9691 | (4546 to 22 470) | 0.22 | 657 | (445 to 1024) | 2.90 | 9035 | (3896 to 20 999) |
| Intensive Care Treatments | 0.44 | 241 | (17 to 1219) | 0.02 | 6 | (2 to 22) | 0.42 | 235 | (13 to 1229) |
| Outpatient Clinic & Emergency Dept. | 6.75 | 1305 | (1087 to 1745) | 3.58 | 584 | (551 to 624) | 3.17 | 721 | (503 to 1189) |
| Day Treatment | 0.60 | 228 | (113 to 628) | 0.33 | 125 | (105 to 148) | 0.27 | 103 | (–13 to 535) |
| Paramedical and supporting staff | 8.13 | 295 | (238 to 377) | 2.91 | 105 | (96 to 116) | 5.22 | 190 | (133 to 271) |
| Other In Hospital Costs | 18.17 | 1765 | (1280 to 2656) | 7.99 | 745 | (636 to 889) | 10.18 | 1020 | (527 to 1960) |
| Total in-hospital costs |  | 43 186 | (35 731 to 58 532) |  | 18 472 | (17 835 to 19 194) |  | 24 714 | (17 497 to 40 561) |
| *Multiple operative procedures could be registered during one operation. | | | | | | | | | |
| SSI; Surgical Site Infection, BCa; Bias-corrected and accelerated, CI; confidence interval | | | | | | | | | |

Table S6. Out-of-hospital costs

|  | **SSI** | | | **Non-SSI** | | | **Difference in** | | |
| --- | --- | --- | --- | --- | --- | --- | --- | --- | --- |
|  | **Mean units** | **Mean cost** | **(BCa 95% CI)** | **Mean units** | **Mean cost** | **(BCa 95% CI)** | **Mean units** | **Mean cost** | **(BCa 95% CI)** |
| Ambulance | 0.20 | 108 | (68 to 163) | 0.07 | 38 | (26 to 54) | 0.12 | 70 | (28 to 131) |
| General practitioner | 2.10 | 65 | (56 to 77) | 1.70 | 52 | (48 to 58) | 0.40 | 13 | (2 to 25) |
| Physiotherapist | 2.92 | 114 | (83 to 157) | 1.71 | 67 | (56 to 79) | 1.21 | 47 | (15 to 92) |
| Speech therapist | 0.14 | 6 | (2 to 15) | 0.01 | 1 | (0 to 1) | 0.13 | 5 | (1 to 15) |
| Psychologist | 0.73 | 72 | (45 to 119) | 0.27 | 27 | (20 to 37) | 0.46 | 46 | (17 to 91) |
| Social worker | 0.22 | 28 | (15 to 51) | 0.03 | 39 | (29 to 52) | –0.08 | –11 | (–27 to 14) |
| Homeopathy | 0.16 | 11 | (3 to 43) | 0.04 | 3 | (1 to 5) | 0.12 | 8 | (0 to 37) |
| Company physician | 0.41 | 49 | (35 to 66) | 0.41 | 49 | (43 to 56) | 0 | 0 | (–15 to 19) |
| Occupational therapist | 0.23 | 6 | (2 to 16) | 0.03 | 1 | (0 to 2) | 0.20 | 5 | (1 to 15) |
| Dietician | 1.49 | 37 | (29 to 48) | 0.50 | 12 | (11 to 14) | 0.99 | 25 | (16 to 36) |
| Housekeeping | 4.41 | 144 | (75 to 290) | 4.17 | 137 | (85 to 233) | 0.24 | 8 | (–96 to 152) |
| Personal care help | 13.00 | 751 | (358 to 1552) | 2.85 | 164 | (105 to 266) | 10.15 | 587 | (205 to 1350) |
| Home nursing | 24.80 | 1860 | (1216 to 2913) | 5.96 | 447 | (300 to 696) | 18.84 | 1413 | (739 to 2463) |
| Total out-of-hospital care costs |  | 3250 | (2263 to 5013) |  | 1036 | (827 to 1333) |  | 2214 | (1232 to 3988) |
| *Mean hours | | | | | | | | | |
| SSI; Surgical Site Infection, BCa; Bias-corrected and accelerated, CI; confidence interval | | | | | | | | | |

Table S7. Mean productivity losses for the entire cohort (including non-workers).

|  | **SSI** | | **Non-SSI** | | **Difference in** | | |
| --- | --- | --- | --- | --- | --- | --- | --- |
|  | **Mean cost** | **(BCa 95% CI)** | **Mean cost** | **(BCa 95% CI)** | **Mean cost** | **(BCa 95% CI)** |  |
| Paid work | 3727 | (2768 to 4845) | 3491 | (2996 to 4035) | 235 | (–852 to 1534) |  |
| Unpaid work | 3400 | (1511 to 11 701) | 971 | (686 to 1659) | 2429 | (499 to 10 935) |  |
| Total productivity losses | 7126 | (4985 to 14 012) | 4462 | (3859 to 5220) | 2664 | (457 to 10 317) |  |
| SSI; Surgical Site Infection, BCa; Bias-corrected and accelerated, CI; confidence interval | | | | | | |  |

Table S8. Mean productivity losses for working individuals only.

|  | **SSI** | | | **Non-SSI** | | | **Difference in** | |  |
| --- | --- | --- | --- | --- | --- | --- | --- | --- | --- |
|  | **n** | **Mean cost** | **(BCa 95% CI)** | **n** | **Mean cost** | **(BCa 95% CI)** | **Mean cost** | **(BCa 95% CI)** |  |
| Paid work | 68 | 11 509 | (9127 to 13 899) | 336 | 9445 | (8275 to 10 637) | 2064 | (–642 to 4693) |  |
| Unpaid work | 88 | 8113 | (3645 to 26 537) | 233 | 3786 | (2711 to 6519) | 4327 | (–456 to 21 218) |  |
| Total productivity losses | 122 | 12 267 | (8790 to 24 665) | 484 | 8379 | (7331 to 9696) | 3887 | (250 to 16 262) |  |
| SSI; Surgical Site Infection, BCa; Bias-corrected and accelerated, CI; confidence interval | | | | | | | | | |

Table S9. Costs per hospital type.

|  | **SSI** | | | **Non-SSI** | | | **Difference** | |
| --- | --- | --- | --- | --- | --- | --- | --- | --- |
|  | **n** | **Mean total cost** | **(BCa 95% CI)** | **n** | **Mean total cost** | **(BCa 95% CI)** | **Mean total cost** | **(BCa 95% CI)** |
| Academic hospital | 105 | 43 295 | (37 588 to 57 142) | 377 | 26 796 | (25 188 to 28 752) | 16 499 | (10 514 to 28 538) |
| Non academic hospital | 105 | 63 830 | (48 977 to 92 513) | 537 | 22 011 | (20 763 to 23 465) | 41 819 | (27 155 to 73 682) |
| SSI; Surgical Site Infection, BCa; Bias-corrected and accelerated, CI; confidence interval | | | | | | | | |

Table S10. High academic in-hospital cost categories.

|  | **SSI (n = 105)** | | | **non-SSI (n = 372)** | | | **Difference** | | |
| --- | --- | --- | --- | --- | --- | --- | --- | --- | --- |
|  | **Mean units** | **Mean cost** | **(BCa 95% CI)** | **Mean units** | **Mean cost** | **(BCa 95% CI)** | **Mean units** | **Mean cost** | **(BCa 95% CI)** |
| General Ward Stay | 16.82 | 15 134 | (13 433 to 17 283) | 7.75 | 7049 | (6631 to 7541) | 9.07 | 8085 | (6356 to 10 361) |
| Operative Procedures* | 1.8 | 9064 | (8146 to 10 179) | 1.45 | 9072 | (8517 to 9668) | 0.35 | -8 | (-1087 to 1207) |
| Intensive Care Unit Stay | 0.67 | 1910 | (754 to 5663) | 0.12 | 388 | (187 to 809) | 0.55 | 1523 | (352 to 5486) |
| SSI; Surgical Site Infection, BCa; Bias-corrected and accelerated, CI; confidence interval | | | | | | | | | |

Table S11. High non-academic in-hospital cost categories.

|  | **SSI (n = 105)** | | | **non-SSI (n = 372)** | | | **Difference** | | |
| --- | --- | --- | --- | --- | --- | --- | --- | --- | --- |
|  | **Mean units** | **Mean cost** | **(BCa 95% CI)** | **Mean units** | **Mean cost** | **(BCa 95% CI)** | **Mean units** | **Mean cost** | **(BCa 95% CI)** |
| General Ward Stay | 19.61 | 17 816 | (14 831 to 22 030) | 7.63 | 6935 | (6549 to 7448) | 11.98 | 10 881 | (7867 to 15 409) |
| Operative Procedures* | 3.04 | 8384 | (7092 to 10 053) | 1.67 | 5699 | (5358 to 6115) | 1.37 | 2685 | (1359 to 4342) |
| Intensive Care Unit Stay | 5.55 | 17 472 | (7312 to 40 474) | 0.29 | 843 | (532 to 1476) | 5.26 | 16 629 | (6248 to 41 508) |
| SSI; Surgical Site Infection, BCa; Bias-corrected and accelerated, CI; confidence interval | | | | | | | | | |

Table S12. Mean health utility scores for different timepoints after surgery.

|  | **SSI** | **Non-SSI** |  |
| --- | --- | --- | --- |
| **Health utility** | **Mean score (SD)** | **Mean score (SD)** | **Mean difference (95% CI)** |
| Day 10 | 0.530 (0.292) | 0.713 (0.221) | -0.183 (-0.221 to -0.146) |
| Day 30 | 0.648 (0.265) | 0.836 (0.181) | 0.188 (-0.219 to -0.158) |
| Day 60 | 0.767 (0.241) | 0.873 (0.175) | 0.105 (-0.134 to -0.077) |
| Day 90 | 0.786 (0.246) | 0.878 (0.180) | 0.091 (-0.120 to -0.062) |
| SSI; Surgical Site Infection, SD; Standard Deviation | | | |

Table S13. Reported QALY in patients treated, specified for procedure indications.

|  | **SSI** | | **Non-SSI** | |  |
| --- | --- | --- | --- | --- | --- |
| **Surgery indication** | **n** | **Mean QALY (SD)** | **n** | **Mean QALY (SD)** | **Mean difference (95% CI)** |
| Benign disease | 84 | 0.159 (0.058) | 203 | 0.194 (0.040) | -0.034 (-0.046 to -0.023) |
| Malignancy | 126 | 0.175 (0.048) | 705 | 0.207 (0.037) | -0.032 (-0.040 to -0.025) |
| SSI; Surgical Site Infection, SD; Standard Deviation | | | | | |
| *For one patient no surgery indication was reported | | | | | |
